# ATP6V1F is a novel prognostic biomarker and potential immunotherapy target for hepatocellular carcinoma

**DOI:** 10.1186/s12920-023-01624-6

**Published:** 2023-08-16

**Authors:** Xinyao Hu, Dan Li, Hua Zhu, Tao Yu, Xiaoxing Xiong, Ximing Xu

**Affiliations:** 1 Cancer Center, Renmin Hospital of Wuhan University, Wuhan University, Wuhan, China; 2 Department of Pharmacy, Renmin Hospital of Wuhan University, Wuhan, China; 3 Department of Neurosurgery, Renmin Hospital of Wuhan University, Wuhan University, Wuhan, China; 4 Department of Oncology, Integrated Traditional Chinese and Western Medicine, The Central Hospital of Wuhan, Tongji Medical College, Huazhong University of Science and Technology, Wuhan, China

**Keywords:** HCC, ATP6V1F, Immunotherapy, TME, Immune cell infiltrates

## Abstract

Hepatocellular carcinoma (HCC) is one of the deadliest malignancies worldwide, with late detection, ineffective treatment and poor overall survival. Immunotherapy, including immune checkpoint inhibitor (ICI) therapy, holds great potential for treatment of HCC. Although some patients respond well to ICIs, many fail to obtain a significant benefit. It is therefore of great interest to find appropriate markers to stratify patient responses to immunotherapy and to explore suitable targets for modulating the TME and immune cell infiltration. ATP6V1F encodes a constituent of vacuolar ATPase (V-ATPase). V-ATPase-mediated acidification of organelles is required for intracellular processes such as zymogen activation, receptor-mediated endocytosis, protein sorting and synaptic vesicle proton gradient generation. In this study, we confirmed for the first time that ATP6V1F is overexpressed in HCC and related to poor prognosis in these patients. We identified that overexpression of ATP6V1F is associated with infiltration of some immune cells and expression of several immune checkpoints. Furthermore, we explored the possible mechanisms of action of ATP6V1F. Finally, we conducted in vitro experiments, including wound healing, Transwell invasion, and apoptosis assays, to verify that ATP6V1F promotes development of HCC by promoting migration and invasion and inhibiting apoptosis of HCC cells. Our findings will contribute to providing precise immunotherapy to patients with HCC.

**Supplementary Information:**

The online version contains supplementary material available at 10.1186/s12920-023-01624-6.

## Introduction

Hepatocellular carcinoma (HCC), the predominant form of primary liver cancer, is the second leading cause of cancer-related death worldwide, with high rates of metastasis and mortality [1–3]. HCC can be treated in early stages by curative therapies such as surgical resection, local area percutaneous ablation and liver transplantation [4]. Unfortunately, due to the insidious onset, limited diagnostic options and rapid progression of HCC, patients tend to be diagnosed at a late stage, resulting in poor prognosis and survival [5]. These patients require palliative systemic therapy, which in the last decade has consisted mainly of the tyrosine kinase inhibitor (TKI) sorafenib, with levatinib, cabozantinib and regorafenib also being approved for use. However, these therapies provide only marginal survival benefits and have considerable toxicity [6].

The immune system is involved in development and progression of cancers. Tumors develop through immune evasion in different ways, such as mediating cytotoxic cell dysfunction or promoting an immunosuppressive microenvironment [7–9]. Immunotherapy for HCC is promising but also very challenging. Overall, use of immune checkpoint inhibitor (ICI) therapies targeting the cytotoxic T lymphocyte (CTLA-4) or programmed cell death ligand 1 (PD-L1)/programmed cell death-1 (PD-1) pathway represents a major breakthrough for a number of cancers. However, the objective response rate for these agents as monotherapy for HCC is only approximately 15–20% [10, 11]. Hence, there is an urgent need to improve the effectiveness of immunotherapy in HCC through biomarker-directed therapy, patient stratification and careful combination selection. In addition, there is strong evidence from clinical data that the immune cell composition of the tumor immune microenvironment (TME) in HCC correlates significantly with overall prognosis and response to therapy [12–15].

ATP6V1F encodes a component of vacuolar ATPase (V-ATPase), which mediates organelle acidification in eukaryotic cells. V-ATPase-dependent organelle acidification is required for intracellular processes such as synaptic vesicle proton gradient generation, receptor-mediated endocytosis, zymogen activation and protein sorting [16]. Expression of ATP6V1s family members has been shown to be significantly related to lymph node metastasis status and clinical cancer stage in patients with renal clear cell carcinoma (KIRC), and ATP6V1s family members may be prognostic markers and therapeutic targets for KIRC [17]. However, the role of ATP6V1F in HCC has not been elucidated.

In our study, by utilizing the GEO, TCGA, HCCDB and HPA databases, we first demonstrate that ATP6V1F expression is upregulated in HCC at both protein and mRNA levels and is related to poor prognosis. Functional enrichment analysis showed that ATP6V1F correlates with multiple tumor-associated signaling pathways. Moreover, the suitability of ICB therapy for HCC patients differed based on ATP6V1F expression level. In vitro experiments demonstrated that ATP6V1F promoted the proliferation and metastasis and inhibited apoptosis of HCC cells. Taken together, our results suggest that ATP6V1F may serve as a novel prognostic and therapeutic stratification marker and a possible target for treatment for HCC.

## Methods

### ATP6V1F expression data download, collation and analysis

Expression data for ATP6V1F were obtained from TCGA (https://tcga.xenahubs.net) (accessed on 09 May 2022) and GEO (GSE102079, including 152 LIHC samples and 14 normal samples) (http://www.ncbi.nlm.nih.gov/geo/) (accessed on 12 May 2022) databases. Gene expression information was processed for normalization by log2 transformation. The "Wilcox.test" approach was used to evaluate differential mRNA expression of ATP6V1F in normal and HCC tissues. The Kruskal‒Wallis test was applied to investigate ATP6V1F expression in different stages of HCC. We used the "ggpubr" R package to plot boxplots. The GEPIA (http://gepia.cancer-pku.cn/index.html) (accessed on 23 May 2022), TIMER (https://cistrome.shinyapps.io/timer/) (accessed on 20 June 2022) and HCCDB (http://lifeome.net/database/hccdb.html) databases were employed to explore expression of ATP6V1F, as we described previously [18].

Next, we examined immunohistochemical (IHC) images of ATP6V1F protein expression in HCC and paracancerous tissues in the Human Protein Atlas (HPA, https://www.proteinatlas.org/) database (accessed 21 June 2022) to investigate differential expression of ATP6V1F.

### Multivariate and univariate Cox regression analyses

Multivariate and univariate Cox regression analyses (*p* < 0.05 for significance) were applied to assess the correlation of ATP6V1F expression with overall survival (OS) and other clinical characteristics (age, race, sex, pTNM stage and grade). A nomogram was developed to determine whether ATP6V1F and such clinicopathological factors are independent contributors to OS. We performed multivariate and univariate Cox hazard regression analyses on the LIHC samples from TCGA using the R package. To predict OS in LIHC patients, we built a validated nomogram using the R 'rms' package and the 'survivor' package. We divided each element into points, summed the points for each argument, and eventually verified the nomogram using calibration curves and the harmonic index (c-index).

### Association analysis of ATP6V1F with prognosis of HCC patients

The GEPIA database was applied to investigate the association of ATP6V1F with OS and DFS in patients with HCC. We also used the database to assess the association between ATP6V1F and other human cancers (accessed on 28 June 2022).

### Analysis of immune infiltration

The TIMER database (accessed on 3 July 2022) was used to investigate the relevance of ATP6V1F expression to infiltration of several immune cells in HCC. In addition, the ESTIMATE approach was applied to assess the relevance of ATP6V1F expression to tumor immune parameters, as previously reported [19].

### Correlation analysis between ATP6V1F expression and immune checkpoints

Next, we extracted expression data for several immune checkpoints, including CTLA4, TIGIT, PDCD1, SIGLEC15, CD274, HAVCR2, LAG3 and PDCD1LG2, in ATP6V1F low and high expression groups (with the median as the cutoff). Multigene correlation plots were generated using the R package. Two-gene correlation plots were drawn with the R package "ggstatsplot". Spearman correlation analysis was performed to describe associations between nonnormally distributed quantitative variables. Patient response to ICI treatment was assessed using the Tumor Immune Dysfunction and Exclusion (TIDE) algorithm.

### GSEA, GO and KEGG pathway enrichment analyses

Enriched KEGG signaling pathway analysis was carried out to determine the potential biological role of ATP6V1F. Gene Ontology (GO) analysis was performed for potential mRNA targets. We used the "ClusterProfiler" R package to cluster the biological process (BP) categories of potential targets. We performed GO and KEGG enrichment analyses of ATP6V1F in LIHC by using LinkedOmics (http://www.linkedomics.org/) (accessed on 1 July 2022) [20]. GSEA was conducted with GSEA software.

### Construction of a protein‒protein interaction (PPI) network

To further examine the role of ATP6V1F in LIHC, the PPI network for ATP6V1F was obtained using STRING (https://cn.string-db.org/) and GeneMANIA (https://genemania.org/) (accessed on 4 July 2022). Module DEGs from the STRING database were ranked based on the intersection of the 11 established topological algorithms described by Chin et al., namely, Degree, Closeness, Betweenness, Radiality, Stress, EcCentricity, BottleNeck, Edge Percolated Component (EPC), Maximum Neighborhood Component (MNC), Density of Maximum Neighborhood Component (DMNC) and Maximal Clique Centrality (MCC) [13].

### Cell lines and culture

The human HCC cell lines Hepg2 and SMMC7721 were obtained from Procell Life Science & Technology Co., Ltd. (Wuhan, China). The cells were cultured in high-glucose Dulbecco's modified Eagle's medium (DMEM) supplemented with 10% fetal bovine serum (FBS; Thermo Fisher Scientific, Inc., Waltham, MA, USA). All cells were grown in a humidified incubator with 5% CO2 at 37 °C.

### Cell transfection

Mimics of ATP6VIF siRNA (5′ to 3’: sense: GCUUAACAAGAACCGCCAUTT, antisense: AUGGCGGUUCUUGUUAAGCTT) and NC (5′ to 3’: sense: UUCUCCGAACGUGUCACGUTT, antisense: ACGUGACACGUUCGGAGAATT) were purchased from Suzhou GenePharma Co., Ltd. Hepg2 and SMMC7721 cells were transfected with ATP6VIF siRNA mimics and NC mimics at a cell density of 60–70% according to the Lipofectamine 6000 kit instructions.

### Wound healing assay

Transfected cells were seeded in 6-well plates and scraped vertically using a 200 μL pipette when the cells reached approximately 90% confluence. Closure of the gap was monitored under the microscope equipped with a digital camera (CK30-SLP; Olympus, Tokyo, Japan) at 0 and 48 h.

### Transwell invasion assays

As we performed previously, seventy microliters of Matrigel (BD Biosciences, San Jose, CA, USA) was used to precoat Transwell chambers at 37 °C for 4 h. Cells were prestarved in serum-free DMEM for 12 h (5 × 104 cells/well) and seeded in the upper chamber of the Transwell plate; DMEM containing 20% FBS was added to the lower chamber. After 24 h of incubation, the remaining cells in the upper chamber were removed with a cotton swab, and the invading cells were fixed with formalin and stained with crystal violet for 15 min. The cells were counted under an inverted microscope (Olympus Corporation) at 200 × magnification [21].

### Apoptosis analysis

Apoptosis was assayed using Annexin V-FITC/Propidium iodide (PI) Apoptosis Detection Kit. Transfected cells were rinsed with ice-cold PBS, resuspended in 100 μL of 1 × binding buffer, stained with 5 μL PI and 5 μL Annexin V-FITC and incubated for 15 min in the dark. Then, another 400 μL binding buffer was added to the mixture before apoptosis was detected using a Beckman cytoFLEX flow cytometer. Analysis of the data obtained was carried out using CytExpert 2.3 software (Beckman Coulter, CA, USA), as we reported previously [22].

### Western blotting

Cell samples were lysed with protease inhibitors and phosphatase inhibitors in RIPA lysis buffer on ice. The supernatant was collected after centrifugation at 12,000 rpm for 20 min. Proteins in each sample were separated with 12% sodium dodecyl sulfate–polyacrylamide gels and transferred to PVDF membranes at 200 mA current (Millipore, NJ, USA). The membranes were blocked with 5% skim milk for 1 h at room temperature and incubated overnight at 4°C with the following primary antibodies: anti-ATP6V1F (1:1000, Proteintech, 17725–1-AP) and anti-GAPDH (1:5000, Proteintech, 60004–1-IG). Anti-rabbit or anti-mouse horseradish peroxidase (HRP)-labeled secondary antibodies were incubated for 60 min at room temperature, and the color was developed using a chemiluminescence kit.

### Statistical analysis

GraphPad Prism 8.0 and R version 4.0.2 were employed for statistical analysis. Experimental data were presented as mean ± SD, and two groups were compared using the Student’s -test. All survival analyses were conducted using the Cox proportional hazards model, the log-rank test and KM analysis. The association of two variables was analyzed by Spearman’s or Pearson’s test. The cutoff to define high or low expression was median expression. *P* value < 0.05 was regarded as significant.

## Results

### ATP6V1F expression in LIHC

**Fig. 1:**
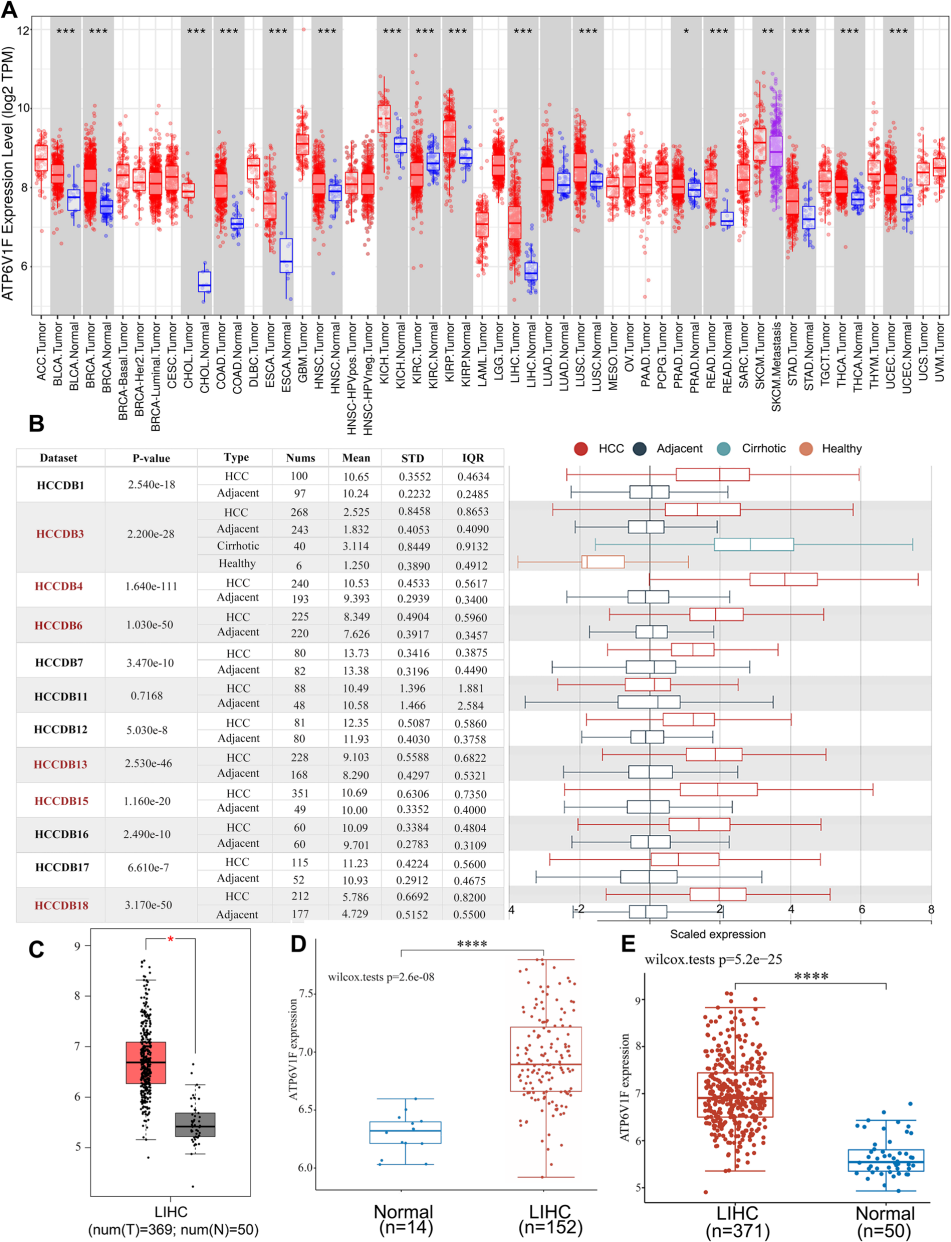
ATP6V1F mRNA expression in HCC and normal tissues. **A** ATP6V1F expression in various tumor and normal tissues. **B** ATP6V1F expression in HCC and adjacent tissues in the HCCDB database. ATP6V1F expression in normal and HCC tissues in GEPIA (**C**), TCGA (**D**), and GEO (**E**) databases. *P < 0.05, ***P < 0.001, ****P < 0.0001

**Fig. 2:**
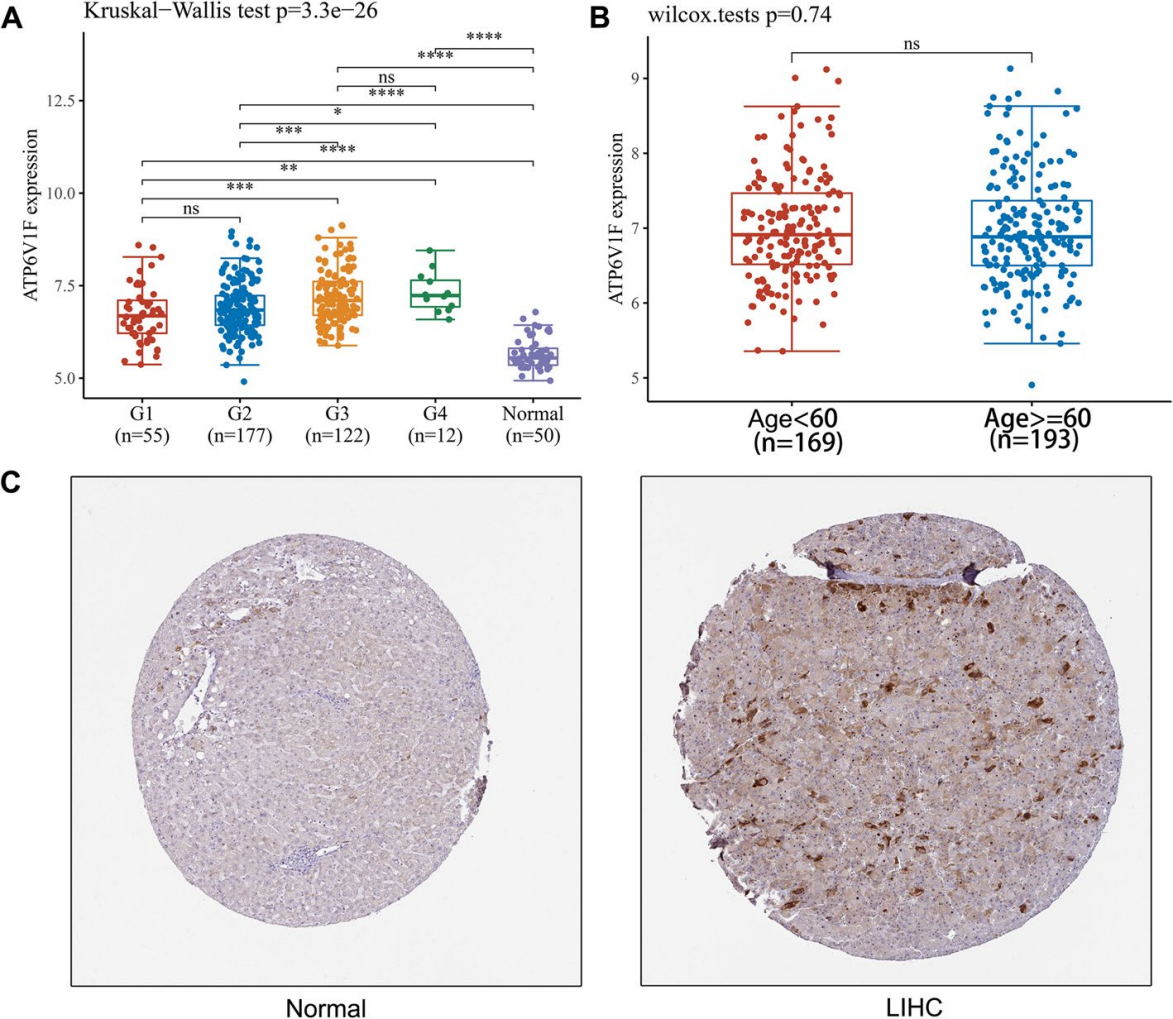
ATP6V1F protein expression in normal and HCC tissues and the correlation between ATP6V1F and tumor stage and patient age. **A** The correlation between ATP6V1F and the tumor stage of HCC. **B** The correlation between ATP6V1F and HCC patient age. (C) ATP6V1F protein IHC in normal and HCC tissues from the HPA database. *P < 0.05, **P < 0.01, ***P < 0.001, ****P < 0.0001, ns: no significant difference

We analyzed ATP6V1F expression in various cancers and paraneoplastic tissues at TIMER and found that ATP6V1F was significantly overexpressed in bladder urothelial carcinoma (BLCA), breast invasive carcinoma (BRCA), cholangiocarcinoma (CHOL), head and neck squamous cell carcinoma (HNSC), esophageal carcinoma (ESCA), kidney chromophobe (KICH), LIHC, kidney renal papillary cell carcinoma (KIRP), lung squamous cell carcinoma (LUSC), rectum adenocarcinoma (READ), prostate adenocarcinoma (PRAD), skin cutaneous melanoma (SKCM), thyroid carcinoma (THCA), stomach adenocarcinoma (STAD) and uterine corpus endometrial carcinoma (UCEC) (Fig. 1A). Data in HCCDB (Fig. 1B) and GEPIA (Fig. 1C) confirmed that ATP6V1F is highly expressed in LIHC. Furthermore, we used TCGA (Fig. 1D, *p* < 0.001) and GEPIA (Fig. 1E, *p* < 0.001) data, and both found that ATP6V1F expression was elevated in LIHC. Next, we explored the association of ATP6V1F with the stage of LIHC and age of patients. The results showed that high expression of ATP6V1F was related to higher LIHC grade, independent of the age of the patient (Fig. 2A, B, Table 1). The association of ATP6V1F expression with LIHC clinicopathological variables is shown in Table 1, indicating that LIHC patients who did not survive tended to have high expression. All results suggest that ATP6V1F is overexpressed in LIHC and may contribute to development of LIHC.

**Table 1:** The relationship of ATP6V1F expression with clinicopathological variables of HCC

	**Clinicopathological variables**	**ATP6V1F-low**	**ATP6V1F-high**	***P*-value**
Status	Alive	132	109	0.312
	Dead	53	77	
Age	Mean (SD)		58.7 (14. 1)	
	Median [MIN, MAX]	60.2 (12.9)	61[61, 85]	0.014
		61 [17, 90]	60	
Gender	FEMALE	61	126	
	MALE	124	81	0.971
	ASIAN	77	6	
	BLACK	11	91	
	WHITE	93	2	
	AMERICAN INDIAN			
pT_stage	T1	102		0.456
	T2	40		
	T2b	1		
	T3	20	25	
	T3a	12	17	
	T3b	2	4	
	T4	6	7	
	TX	1		
	T2a		1	
pN_stage	N0	123	129	0.249
	NX	62	52	
	N1			
pM_stage	M0	128	138	0.381
	M1	2	2	
	MX	55	46	
pTNM_stage	I	95	76	0.556
	II	40	46	
	III	2	1	
	IIIA	27	38	
	IIIB	2	6	
	IIIC	3	6	
	IV	1	1	
	IVB	1	1	
	IVA		1	
Grade	G1	35	20	0.362
	G2	97	80	
	G3	48	74	
	G4	3	9	
New_tumor_ event_type	Primary	7		0.003
	Recurrence	76		
	Non-radiation	128		0.267
Radiation_therapy	Radiation	1		0.535
	Neoadjuvant	1	1	
	No neoadjuvant	184	185	1
	Chemotherapy	15	14	
	Chemotherapy:	1		
	Hormone Therapy			
	Chemotherapy:Targeted	1	1	
	Molecular therapy			
	Other. specify in notes	1		
	Ancillary		1	
	Chemotherapy:Hormone Therapy:Other specify in notes	1		
	Targeted Molecular therapy		5	1

Differential ATP6V1F expression at the protein level in LIHC and normal liver tissues was investigated by using the HPA database. We investigated whether the ATP6V1F protein is also significantly elevated in LIHC (Fig. 2C).

### ATP6V1F prognostic value in LIHC

**Fig. 3:**
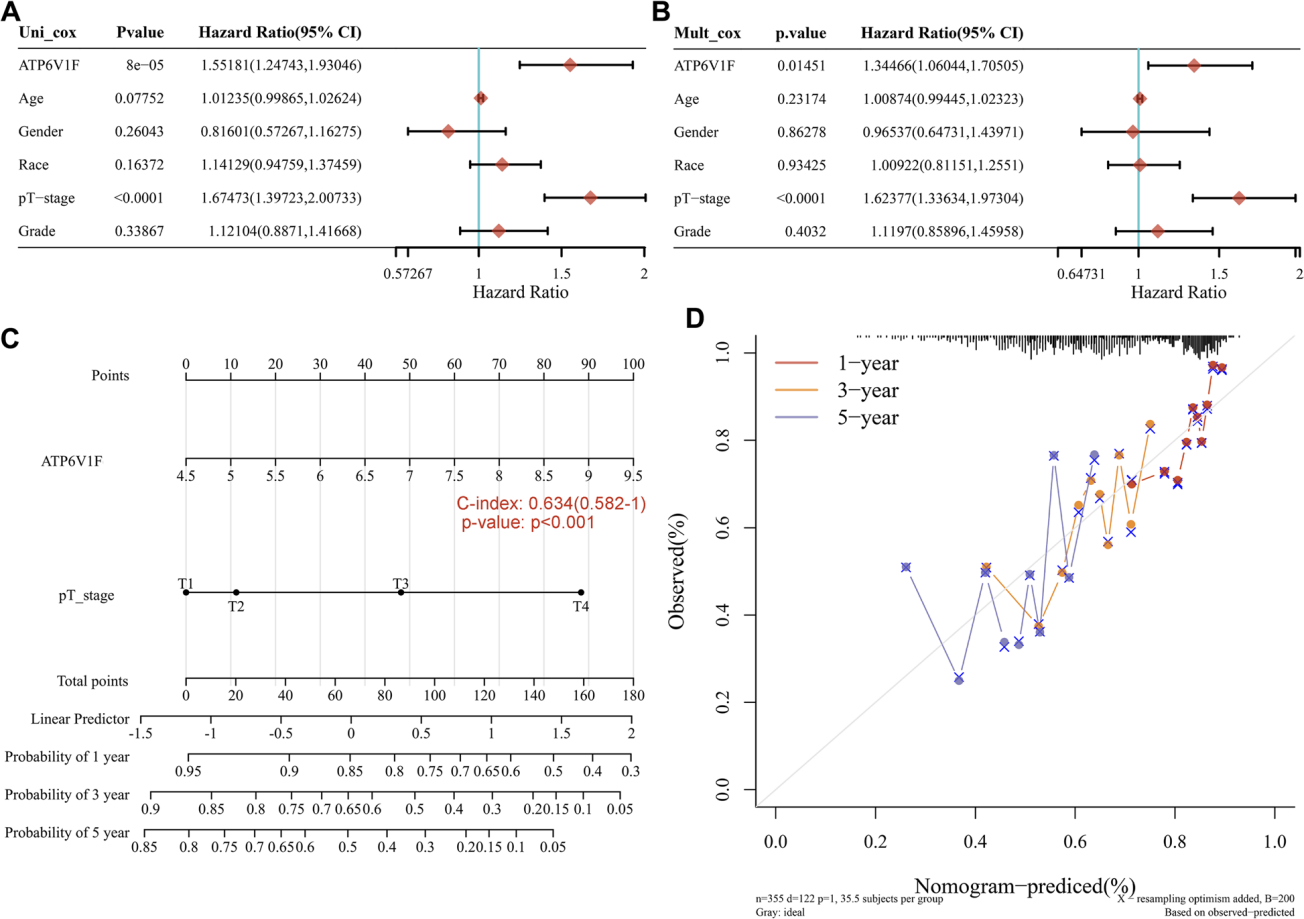
The prognostic value of ATP6V1F in HCC. **A** Univariate and (**B**) multifactorial Cox analyses of ATP6V1F and other clinical factors in LIHC. **C** A nomogram and (**D**) calibration curves of ATP6V1F and pT-stage were established to predict 1-, 3-, and 5-year OS in HCC patients. Circles and crosses are observed mortality rates and 95% CIs

Both univariate (Fig. 3A, *P* < 0.001) and multivariate (Fig. 3B, *P* = 0.01451) Cox regression analyses showed that ATP6V1F expression may be an independent prognostic factor in LIHC. The nomogram showed that higher ATP6V1F expression and pathological T stage were associated with higher points and lower survival rates at 1, 3, and 5 years, suggesting that ATP6V1F can serve as an independent factor affecting patient outcome (Fig. 3C, D, *P* < 0.001).

**Fig. 4:**
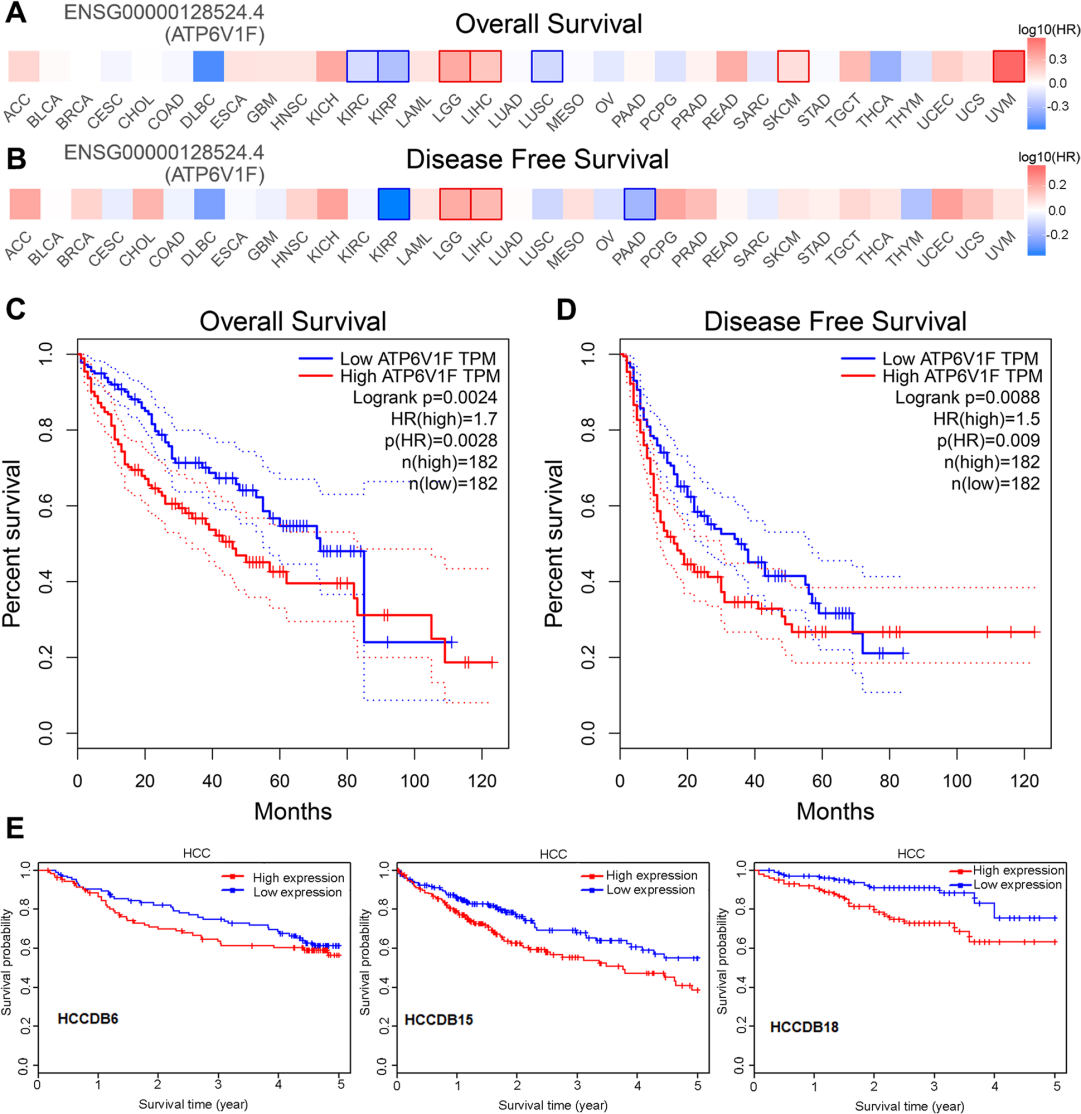
The correlation between AFP6V1F and the OS and DFS of HCC. The relationship of ATP6V1F with the OS (**A**) and DFS (**B**) of pancancer. The correlation between AFP6V1F and OS (**C**) and DFS (**D**) in HCC. **E** The correlation of ATP6V1F with the OS of HCC in the HCCDB database

We further mined the GEPIA database for the association of ATP6V1F with OS and disease-free survival (DFS) in cancer patients. We found that ATP6V1F expression was negatively related to OS and DFS in patients with KIRP but positively related to OS and DFS in LGG (brain lower grade glioma) and LIHC (Fig. 4A, B), suggesting that ATP6V1F plays different roles in different tumors. For LIHC patients, high ATP6V1F predicted poorer OS (Fig. 4C, *P* = 0.0028) and DFS (Fig. 4D, *P* = 0.009), a trend also observed with HCCDB data (Fig. 4E).

### ATP6V1F is associated with immune cell infiltration in LIHC

**Fig. 5:**
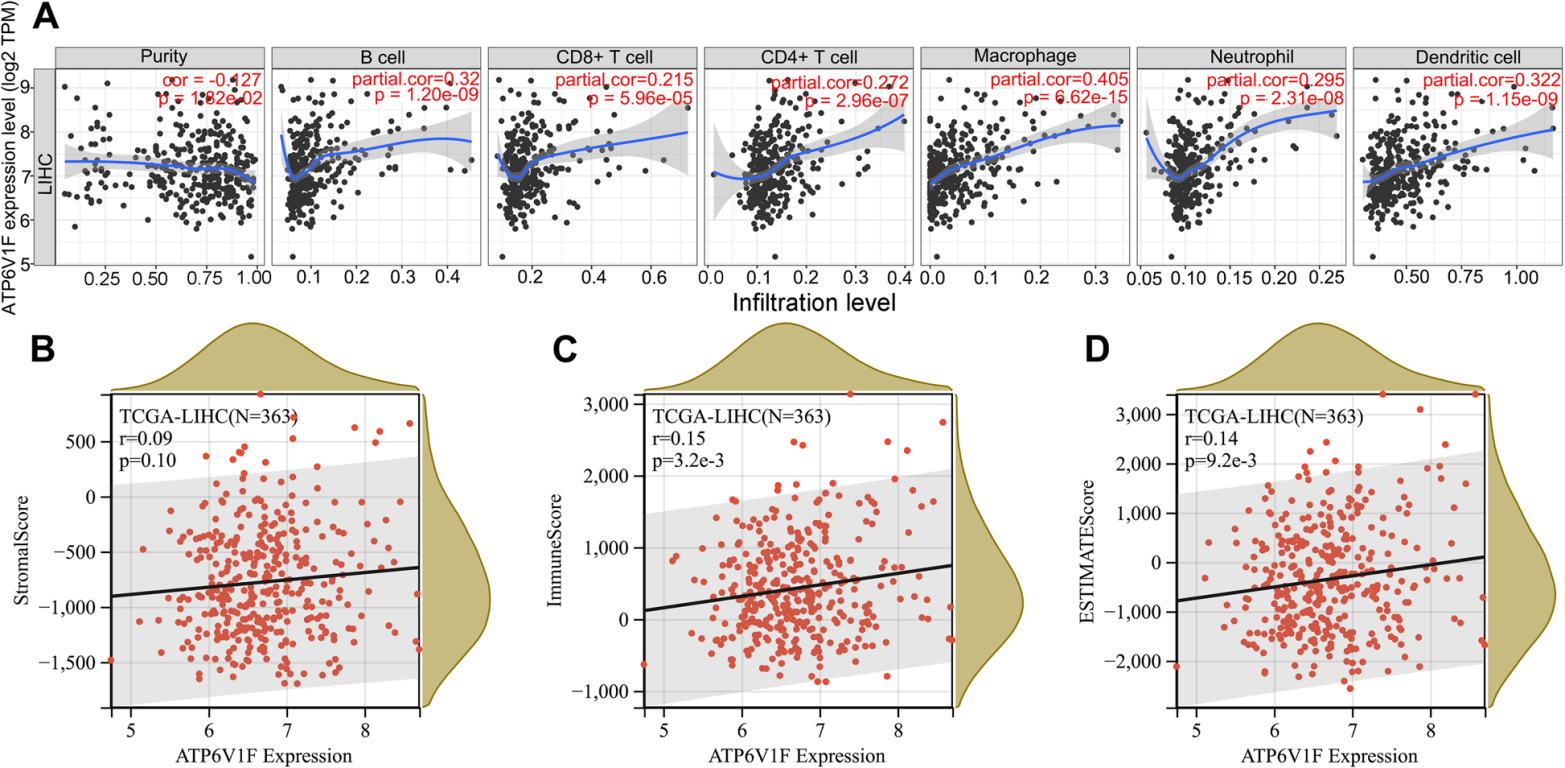
ATP6V1F is related to infiltration of several immune cells in the TME of HCC. **A** ATP6V1F is related to infiltration of B cells, CD4 + T cells, CD8 + T cells, macrophages, dendritic cells and neutrophils. **B** ATP6V1F related to the ESTIMATE score and immune score

We explored the correlation of ATP6V1F with immune cell infiltration in LIHC in the TIMER database, revealing that ATP6V1F expression promoted infiltration of B cells (*p* < 0.001), CD4 + T cells (*p* < 0.001), CD8 + T cells (*p* < 0.001), macrophages (*p* < 0.001), dendritic cells (*p* < 0.001) and neutrophils (*p* < 0.001) (Fig. 5A). Moreover, ATP6V1F expression was positively related to the immune score (*p* = 0.0032) and ESTIMATE score (*p* = 0.0092) in LIHC (Fig. 5C, D) but was not associated with the stromal score (Fig. 5B).

### ATP6V1F expression is associated with response to immunotherapy in LIHC

**Fig. 6:**
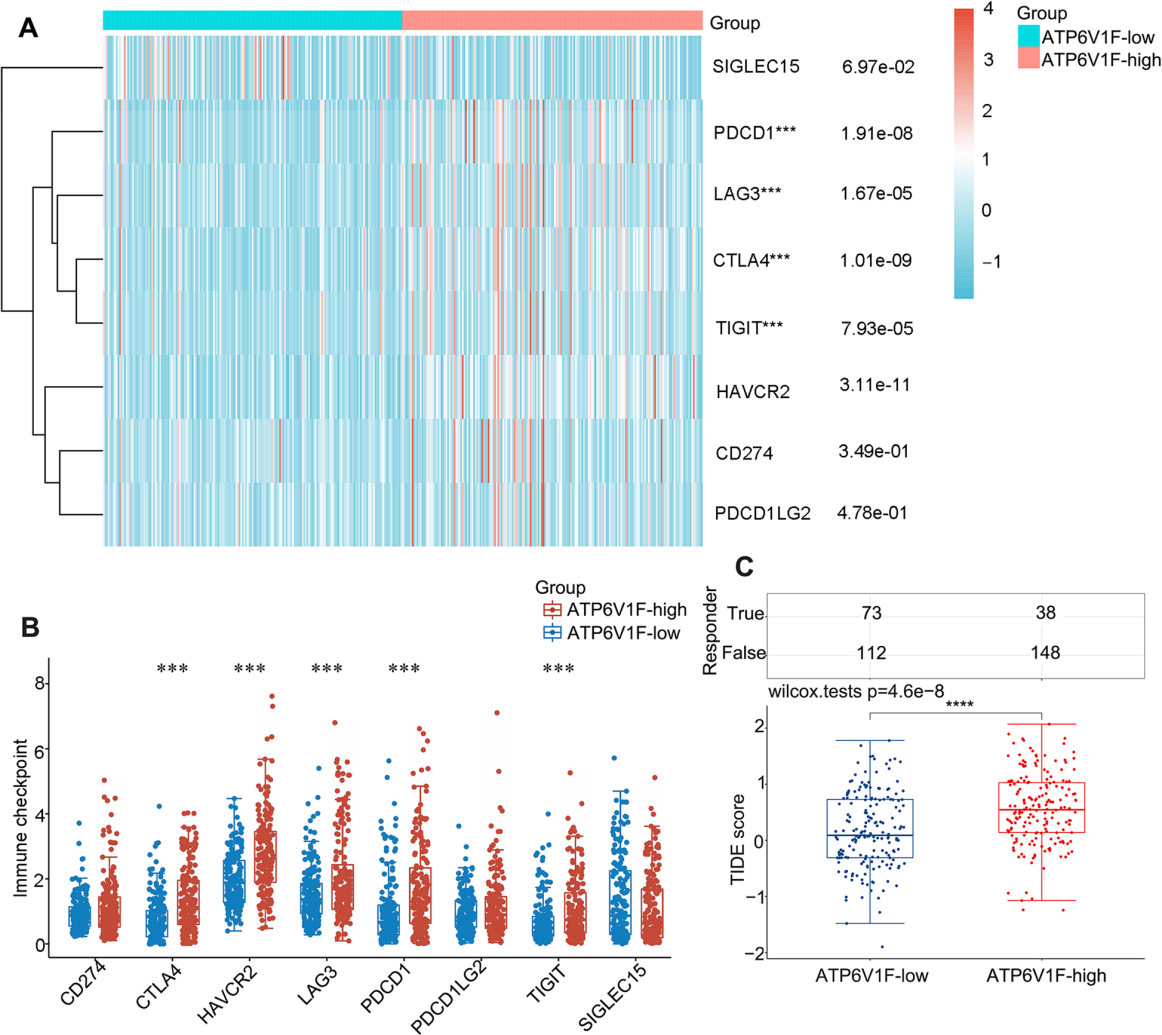
ATP6V1F is related to immune checkpoint expression and patient response to ICB. **A**, **B** ATP6V1F is coexpressed with several immune checkpoints. **C** TIDE scores in ATP6V1F-low and ATP6V1F-high groups in HCC. **P < 0.01, ***P < 0.001

**Fig. 7:**
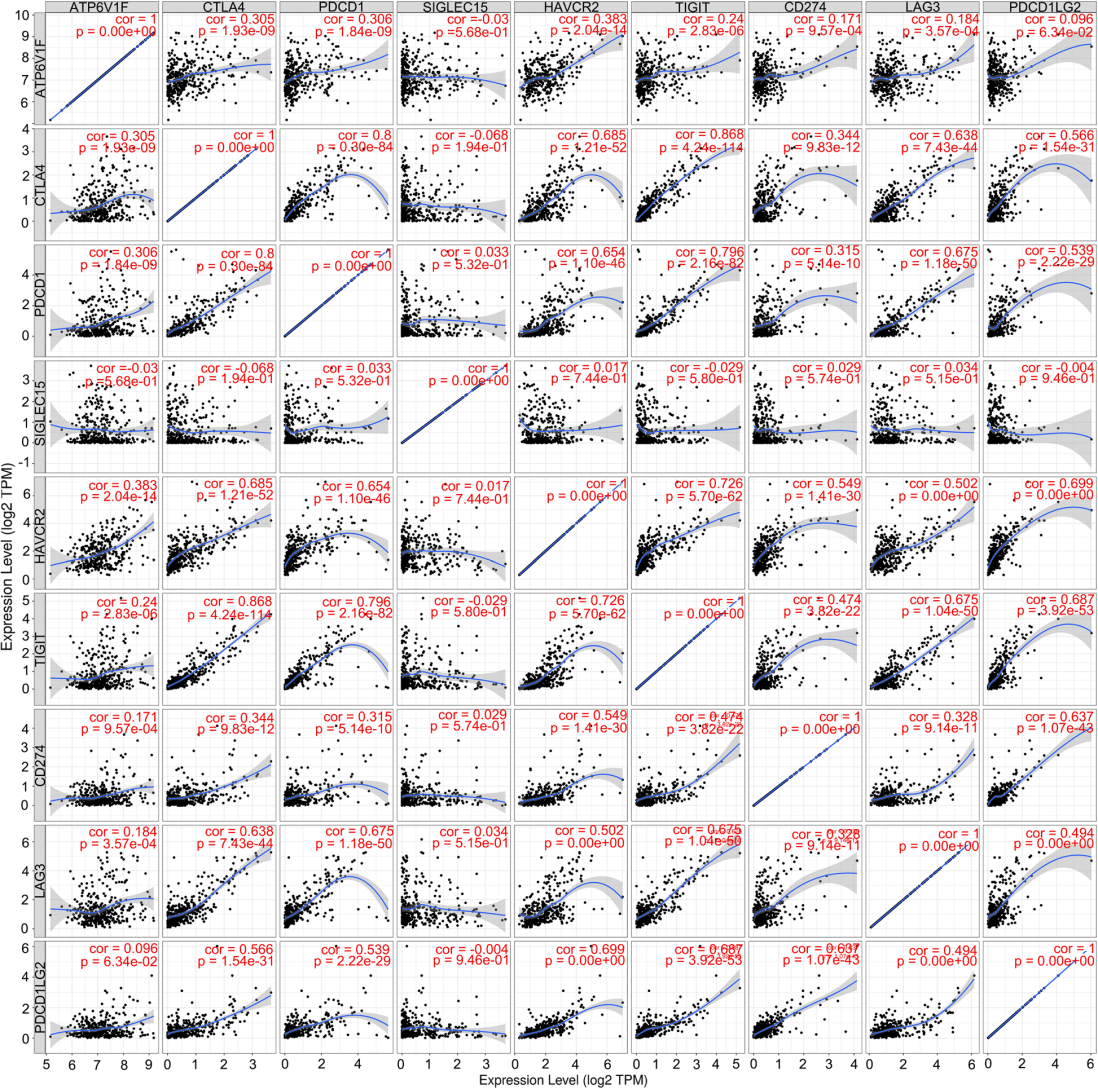
ATP6V1F correlates with immune checkpoint expression

Moreover, we found ATP6V1F to be coexpressed with several immune checkpoints, including PDCD1 (*p* < 0.001), CTLA4 (*p* < 0.001), LAG3 (*p* < 0.001), TIGIT (*p* < 0.001), and HAVCR2 (*p* < 0.001) (Fig. 6A, B). Additionally, patients with higher expression of ATP6V1F had a higher TIDE score (Fig. 6C, Figure S1). We validated the correlation between ATP6V1F expression and immune checkpoint expression in the TIMER database and found that the correlation coefficients between ATP6V1F expression and the expression of CTLA4, PDCD1, HAVCR2, CD274 and LAG3 were 0.305, 0.306, 0.383, 0.184 and 0.305, respectively (Fig. 7). We speculate that ATP6V1F has potential as a stratification marker for immunotherapy.

### The potential function of ATP6V1F in LIHC

**Fig. 8:**
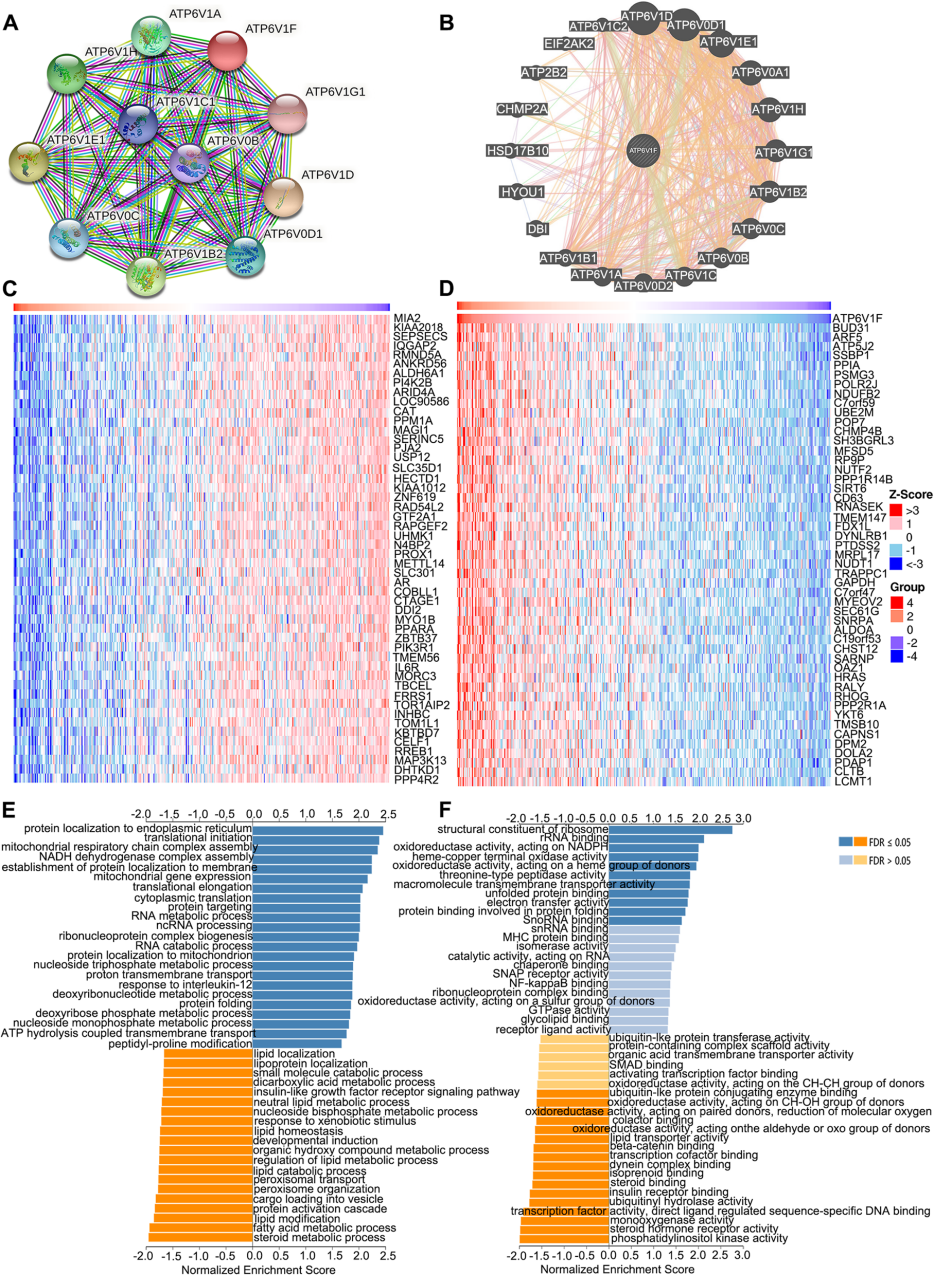
The potential function of ATP6V1F. PPI networks constructed using the (**A**) STRING and (**B**) GeneMANIA databases. The top 50 genes negatively (**C**) and positively (**D**) related to TMCO3 in LIHC. (E) GO BP analysis of ATP6V1F. **F** KEGG pathway analysis of ATP6V1F [23–25]

We first constructed a PPI network using the STRING and GeneMANIA databases. The results showed that ATP6VIF may function by interacting with ATP6V1A, ATP6V1D, ATP6V0D1, ATP6V1E1, ATP6V1H, ATP6V1B2, ATP6V0C, ATP6V0B and ATP6V1G1 (Fig. 8A, B). The top 50 genes most negatively and positively related to ATP6V1F expression are illustrated in Fig. 8C and Fig. 8D, respectively. The top 10 genes most negatively related to ATP6V1F were MIA2, KIAA2018, SEPSECS, IQGAP2, RMND5A, ANKRD56, ALDH6A1, PI4K2B, ARID4A and LOC90586 (Fig. 8C). The top 10 genes most positively related to ATP6V1F were BUD31, ARF5, ATP5J2, SSBP1, PPIA, PSMG3, POLR2J, NDUFB2, C7orf59 and UBE2M (Fig. 8D). In addition, more genes related to ATPV1F were obtained in the STRING database, and they imported into Cytoscape for 11 topological analyses (Figure S2). The top ranked and overlapping hub genes according to 11 topological algorithms are listed in Table S1.

**Fig. 9:**
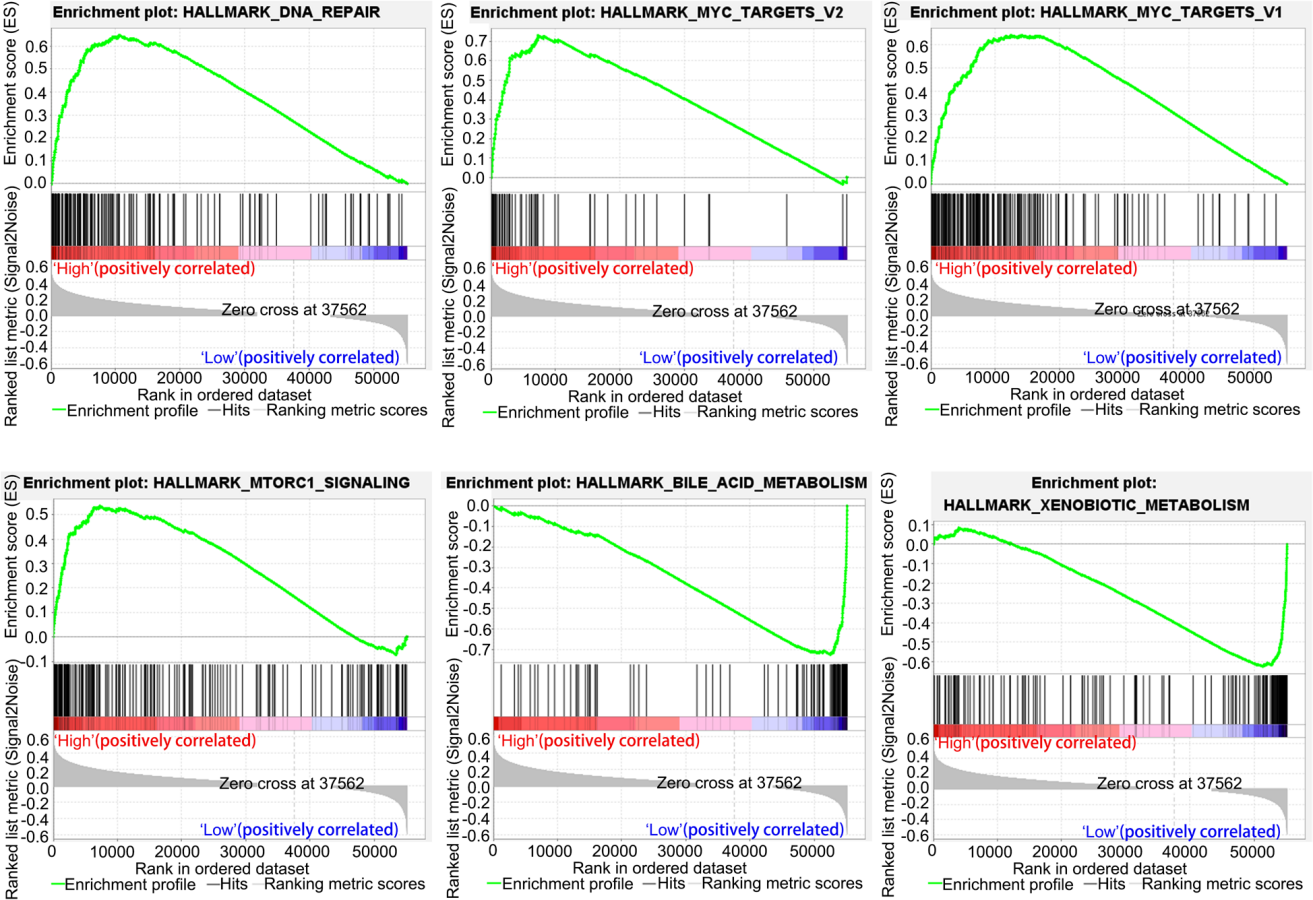
GESA pathway enrichment analysis of ATP6V1F

**Fig. 10:**
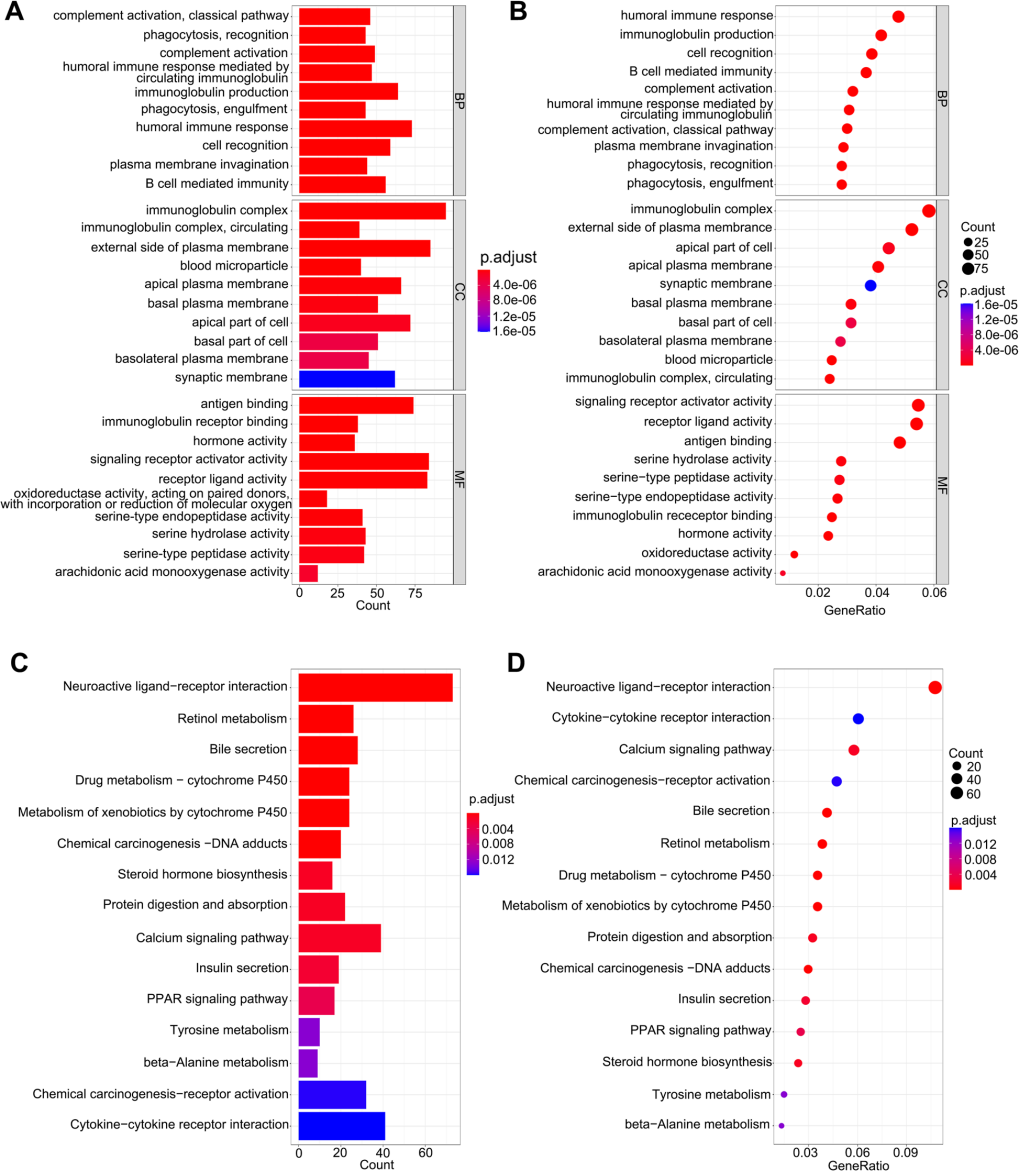
GO analysis and KEGG pathway enrichment analysis of ATP6V1F by R software. **A**, **B** GO BP, CC, and MF analyses of ATP6V1F. **C**, **D** KEGG pathway enrichment analysis of ATP6V1F

Subsequently, we performed KEGG pathway enrichment analysis and GO analysis using the Linkedomics database to explore the possible mechanisms of ATP1V1F and related pathways. GO analysis of biological process (BF) showed that ATP1V1F is involved in protein localization to endoplasmic reticulum, translational initiation, NADH dehydrogenase complex assembly, mitochondrial respiratory chain complex assembly, establishment of protein localization to membrane, mitochondrial gene expression, translational elongation, and cytoplasmic translation, among others (Fig. 8E). KEGG pathway analysis indicated that ATP6V1F may act through processes of the structural constituent of ribosome, oxidoreductase activity, acting on NAD(P)H, rRNA binding, oxidoreductase activity, acting on a heme group of donors, heme-copper terminal oxidase activity and acting on a heme group of donor’s oxidoreductase activity (Fig. 8F). GESA analysis revealed that high ATP1VF expression is positively associated with the MYC target V2, DNA repair, and MTORC1 signaling pathways and MYC target V1 but negatively to the bile acid metabolism and xenobiotic metabolism pathways (Fig. 9). Moreover, we further analyzed potential pathways involved in ATP6V1F using R software. GO BP analysis confirmed the possible participation of ATP6V1F in biological processes such as phagocytosis, recognition, complement activation, the classical pathway, the humoral immune response mediated by circulating immunoglobulin, complement activation, immunoglobulin production and the humoral immune response. GO cellular component (CC) analysis indicated that ATP6V1F may perform functions in the immunoglobulin complex, external side of the plasma membrane immunoglobulin complex, and circulating and apical parts of the cell. GO molecular function analysis showed that ATP6V1F functions through antigen binding, immunoglobulin receptor binding, signaling receptor activator activity and receptor ligand activity (Fig. 10A, B). KEGG enrichment analysis also showed the relationship between ATP6V1F expression and the calcium signaling pathway, neuroactive ligand‒receptor interaction, retinol metabolism, bile secretion and drug metabolism—cytochrome P450 (Fig. 10C, D).

### ATP6V1F influences migration, invasion, and apoptosis of HCC cells

**Fig. 11:**
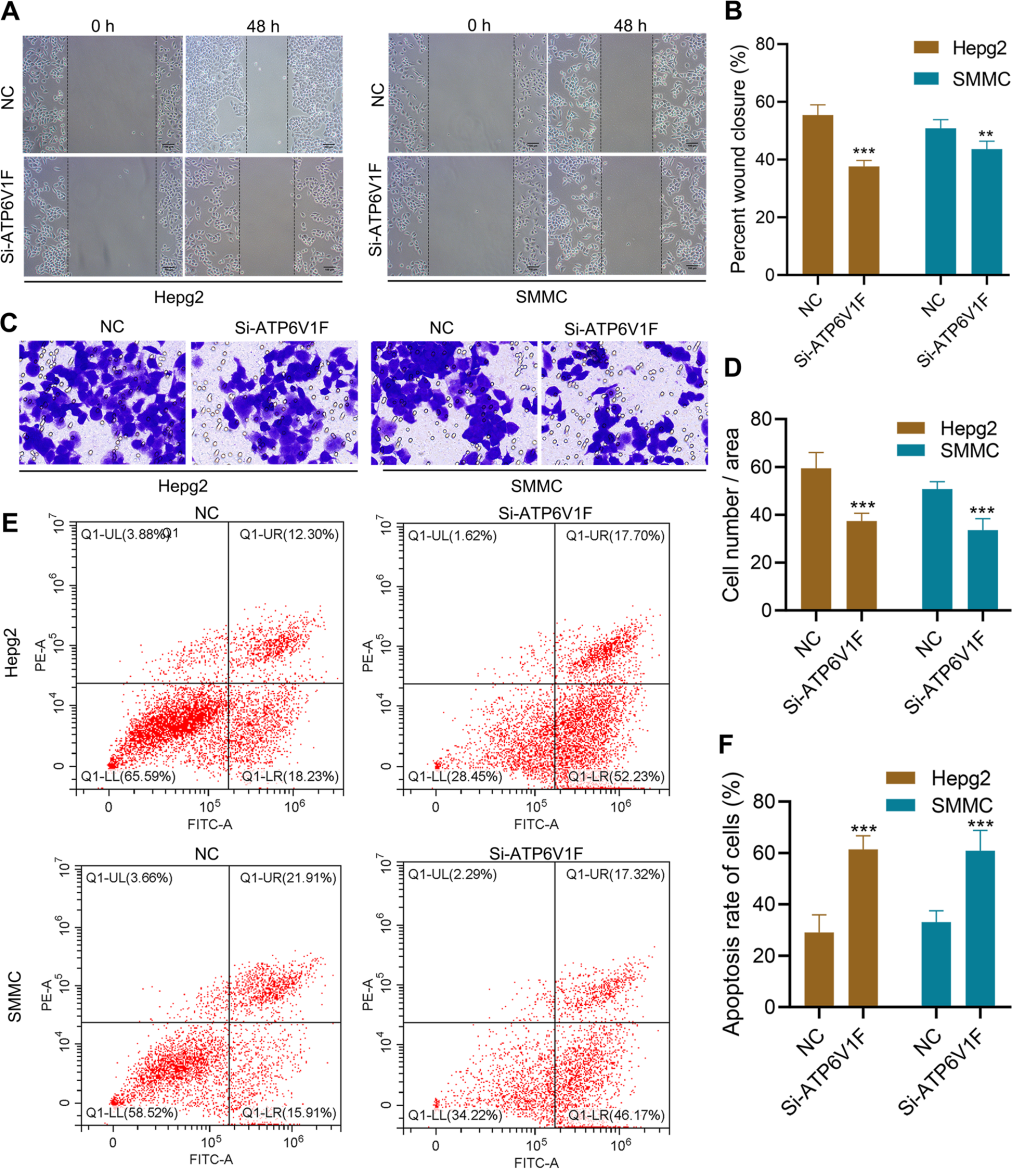
ATP6V1F influences the migration, invasion, and apoptosis of HCC cells in vitro. **A**, **B** Wound healing assay shows that ATP6V1F enhances migration of Hepg2 and SMMC7721 cells. **C**, **D** Transwell invasion assay shows that ATP6V1F enhances invasion of Hepg2 and SMMC7721 cells. (E, F) ATP6V1F inhibits apoptosis in Hepg2 and SMMC7721 cells

A series of in vitro experiments was conducted to validate the role of ATP6V1F. We verified that ATP6V1F protein expression was successfully knocked down by si-ATP6V1F in HCC cells using western blot assay (Figure S3). Wound healing assays showed that knockdown of ATP6V1F decreased the rate of wound healing, suggesting that ATP6V1F promotes cell migration (Fig. 11A, B). A Transwell invasion assay revealed that knockdown of ATP6V1F inhibited invasion of HCC cells (Fig. 11C, D), and ATP6V1F inhibited HCC cell apoptosis (Fig. 11E, F). The above results suggest that ATP6V1F promotes development of HCC by promoting migration and invasion of HCC cells and inhibiting their apoptosis.

## Discussion

HCC is one of the world's deadliest malignancies. Due to the late presentation of symptoms, HCC is usually diagnosed at an advanced stage, with very limited treatment options, leading to ineffective interventions and poor prognosis [26, 27]. ICIs have emerged as a promising treatment for a number of solid and liquid tumors, such as melanoma, renal cell carcinoma (RCC), uroepithelial carcinoma, non-small cell lung cancer (NSCLC) and Hodgkin's lymphoma (HL) [28–30]. The US FDA has also approved the combination of bevacizumab and atezolizumab (PD-L1 inhibitor) as first-line therapy for patients with unresectable HCC, heralding the great potential of ICIs for treatment of HCC [31]. Although some patients respond well to ICIs, many do not achieve a significant benefit, and others may show unexpected and paradoxical deterioration [32, 33]. It is therefore of great interest to identify appropriate markers to stratify patient response to ICIs and to explore the suitable targets to regulate the TME and immune cell infiltration.

Possible therapeutic targets for HCC have been reported in previous studies, such as RAB7A [34], RRM2 [35], and CKLF[36]. In our study, we first confirmed that both protein and mRNA levels of ATP6V1F in HCC were evaluated by different methods. Subsequently, we found that elevated ATP6V1F predicted higher grade HCC and poor outcome. Additionally, multivariate and univariate analyses corroborated that ATP6VIF is an independent prognostic factor for HCC and predictive of 1-, 3- and 5-year survival in these patients. Next, we explored the relationship between ATP6V1F and immune cell infiltration in the TME, as well as immune checkpoint expression, to explore the potential of ATP6V1F as a biomarker for immunotherapy stratification of patients and target of immunotherapy. The results showed that ATP6V1F promotes infiltration of a number of immune cells, including B cells, CD4 + T cells, CD8 + T cells, macrophages, dendritic cells and neutrophils. Therefore, targeting ATP6V1F may be a promising approach to enhance the immune response to tumors. Moreover, expression of ATP6V1F was positively related to the ESTIMATE score and immune score, and ATP6V1F was coexpressed with several immune checkpoints, including PDCD1, LAG3, CTLA4, TIGIT, and HAVCR2. For example, patients with higher expression of ATP6V1F had a higher TIDE score. The results from the TIMER website also showed ATP6V1F expression to be positively related to expression of CTLA4, PDCD1, HAVCR2, LAG3 and CD274. The findings suggest that HCC patients with high ATP6V1F expression respond better to ICI treatment.

Furthermore, we explored the potential function of ATP6V1F by studying the genes and pathways associated with it. The results indicated that ATP6VIF may act by interacting with ATP6V1A, ATP6V1D, ATP6V0D1, ATP6V1E1, ATP6V1H, ATP6V1B2, ATP6V0C, ATP6V0B and ATP6V1G1. The top 10 genes most negatively associated with ATP6V1F were MIA2, KIAA2018, SEPSECS, IQGAP2, RMND5A, ANKRD56, ALDH6A1, PI4K2B, ARID4A and LOC90586; the top 10 genes most positively related were BUD31, ARF5, ATP5J2, SSBP1, PPIA, PSMG3, POLR2J, NDUFB2, C7orf59 and UBE2M. These proteins may interact with ATP6V1F to exert a tumorigenic effect. GO analysis revealed that ATP6V1F may participate in biological processes such as complement activation, the classical pathway, complement activation, immunoglobulin production, the humoral immune response, and the humoral immune response mediated by circulating immunoglobulin. Moreover, ATP6V1F may perform functions in the immunoglobulin complex, external side of the plasma membrane and apical part of the cell, immunoglobulin complex, and circulation. Based on KEGG enrichment analysis, ATP6V1F expression is related to the calcium signaling pathway, neuroactive ligand‒receptor interaction, retinol metabolism, bile secretion and drug metabolism—cytochrome P450. Finally, we performed wound healing assays, Transwell invasion assays and flow cytometry assays to verify in vitro that ATP6V1F promotes development of HCC by promoting migration and invasion of and inhibiting apoptosis of HCC cells.

Despite our in-depth analysis of the potential of ATP6V1F as a prognostic marker and immunotherapeutic target for HCC, there are still some limitations in this study. First, animal experiments are still needed to validate the role of ATP6V1F in vivo. Second, due to the complexity of the immune microenvironment, single-cell sequencing, flow sorting, and standardized clinical trials should be performed to validate the relevance of ATP6V1F in the immune microenvironment before targeting ATP6V1F to assist in immunotherapy. In addition, stratified screening of patient responsiveness is a crucial step prior to implementation of ATP6V1F-targeted therapy.

## Conclusion

In summary, we identified for the first time that ATP6V1F is overexpressed in HCC and related to its poor prognosis. We investigated the association of ATP6V1F with immune cell infiltration in HCC and coexpression of ATP6V1F with immune checkpoints, confirming the potential of ATP6V1F as a marker for immunotherapeutic stratification and a target for immunotherapy. Finally, we explored the possible mechanisms of action of ATP6V1F and verified the effect of ATP6V1F through in vitro experiments. Our findings will help to provide precise immunotherapy for HCC patients.

### Supplementary Information

**Additional file 1: Figure S1.** The PPI network of ATP6V1F constructed using the STRING database. **Figure S2.** TIDE scores in ATP6V1F-low and ATP6V1F-high groups in HCC. Set the top ¼ as high expression group and the bottom ¼ as low expression group. ****P < 0.0001. G1: low expression group, G2: high expression group. **Figure S3.** Western blot assays verified that ATP6V1F protein expression was successfully knocked down by si-ATP6V1F in Hepg2 and SMMC7721 cells (A). Statistical results of western blot assays (B). ***P < 0.001.

**Additional file 2:** **Table S1.** The top ranked and overlapping hub genes according to 11 topological algorithms in the PPI networks.

## Data Availability

The datasets presented in this study can be found in the online repositories TCGA (https://tcga.xenahubs.net) and GEO (https://www.ncbi.nlm.nih.gov/geo/, GSE102079). The names of the repositories/repositories and accession number(s) can also be found in the article.

## References

[1] Hu X, Zhu H, Shen Y, Zhang X, He X, Xu X (2021). The role of non-coding RNAs in the Sorafenib resistance of hepatocellular carcinoma. Front Oncol.

[2] Gentile D, Donadon M, Lleo A, Aghemo A, Roncalli M, di Tommaso L (2020). Surgical treatment of hepatocholangiocarcinoma: a systematic review. Liver Cancer.

[3] Ioannou GN (2021). Epidemiology and risk-stratification of NAFLD-associated HCC. J Hepatol.

[4] Ruf B, Heinrich B, Greten TF (2021). Immunobiology and immunotherapy of HCC: spotlight on innate and innate-like immune cells. Cell Mol Immunol.

[5] Li D, Yu T, Han J, Xu X, Wu J, Song W (2021). Prognostic value and immunological role of KIFC1 in hepatocellular carcinoma. Front Mol Biosci.

[6] Sangro B, Sarobe P, Hervás-Stubbs S, Melero I (2021). Advances in immunotherapy for hepatocellular carcinoma. Nat Rev Gastroenterol Hepatol.

[7] Foerster F, Gairing SJ, Ilyas SI, Galle PR (2022). Emerging immunotherapy for HCC: a guide for hepatologists. Hepatology.

[8] Yu T, Li D, Zeng Z, Xu X, Zhang H, Wu J (2022). INSC Is down-regulated in colon cancer and correlated to immune infiltration. Front Genet.

[9] Xia L, Oyang L, Lin J, Tan S, Han Y, Wu N (2021). The cancer metabolic reprogramming and immune response. Mol Cancer.

[10] Zongyi Y, Xiaowu L (2020). Immunotherapy for hepatocellular carcinoma. Cancer Lett.

[11] Cheng AL, Hsu C, Chan SL, Choo SP, Kudo M (2020). Challenges of combination therapy with immune checkpoint inhibitors for hepatocellular carcinoma. J Hepatol.

[12] Kurebayashi Y, Ojima H, Tsujikawa H, Kubota N, Maehara J, Abe Y (2018). Landscape of immune microenvironment in hepatocellular carcinoma and its additional impact on histological and molecular classification. Hepatology.

[13] Zhu H, Hu X, Gu L, Jian Z, Li L, Hu S (2021). TUBA1C is a prognostic marker in low-grade glioma and correlates with immune cell infiltration in the tumor microenvironment. Front Genet.

[14] Zhu H, Hu X, Ye Y, Jian Z, Zhong Y, Gu L (2021). Pan-cancer analysis of PIMREG as a biomarker for the prognostic and immunological role. Front Genet.

[15] Lee YH, Tai D, Yip C, Choo SP, Chew V (2020). Combinational immunotherapy for hepatocellular carcinoma: radiotherapy, immune checkpoint blockade and beyond. Front Immunol.

[16] Du YJ, Hou YL, Hou WR (2012). Cloning and overexpression of an important functional gene ATP6V1F encoding a component of vacuolar ATPase from the Giant Panda (Ailuropoda melanoleuca). Mol Biol Rep.

[17] Li X, Li H, Yang C, Liu L, Deng S, Li M (2020). Comprehensive analysis of ATP6V1s family members in renal clear cell carcinoma with prognostic values. Front Oncol.

[18] Zhu H, Hu X, Feng S, Jian Z, Xu X, Gu L (2022). The Hypoxia-related gene COL5A1 is a prognostic and immunological biomarker for multiple human tumors. Oxid Med Cell Longev.

[19] Hu X, Zhu H, Chen B, He X, Shen Y, Zhang X (2022). The oncogenic role of tubulin alpha-1c chain in human tumours. BMC Cancer.

[20] Vasaikar SV, Straub P, Wang J, Zhang B (2018). LinkedOmics: analyzing multi-omics data within and across 32 cancer types. Nucleic Acids Res.

[21] Liu X, Hu Y, Li C, Chen J, Liu X, Shen Y (2023). Overexpression of YEATS2 remodels the extracellular matrix to promote hepatocellular carcinoma progression via the PI3K/AKT pathway. Cancers (Basel).

[22] Xu Y, He X, Deng J, Xiong L, Chen B, Chen J (2022). ROS-related miRNAs regulate immune response and chemoradiotherapy sensitivity in hepatocellular carcinoma by comprehensive analysis and experiment. Oxid Med Cell Longev.

[23] Kanehisa M, Goto S (2000). KEGG: kyoto encyclopedia of genes and genomes. Nucleic Acids Res.

[24] Kanehisa M, Furumichi M, Sato Y, Kawashima M, Ishiguro-Watanabe M (2023). KEGG for taxonomy-based analysis of pathways and genomes. Nucleic Acids Res.

[25] Kanehisa M (2019). Toward understanding the origin and evolution of cellular organisms. Protein Sci.

[26] Xing R, Gao J, Cui Q, Wang Q (2021). Strategies to improve the antitumor effect of immunotherapy for hepatocellular carcinoma. Front Immunol.

[27] Couri T, Pillai A (2019). Goals and targets for personalized therapy for HCC. Hepatol Int.

[28] Kennedy LB, Salama AKS (2020). A review of cancer immunotherapy toxicity. CA Cancer J Clin.

[29] Bagchi S, Yuan R, Engleman EG (2021). Immune checkpoint inhibitors for the treatment of cancer: clinical impact and mechanisms of response and resistance. Annu Rev Pathol.

[30] Giannone G, Ghisoni E, Genta S, Scotto G, Tuninetti V, Turinetto M (2020). Immuno-metabolism and microenvironment in cancer: key players for immunotherapy. Int J Mol Sci.

[31] Galle PR, Finn RS, Qin S, Ikeda M, Zhu AX, Kim TY (2021). Patient-reported outcomes with atezolizumab plus bevacizumab versus sorafenib in patients with unresectable hepatocellular carcinoma (IMbrave150): an open-label, randomised, phase 3 trial. Lancet Oncol.

[32] Harkus U, Wankell M, Palamuthusingam P, McFarlane C, Hebbard L. Immune checkpoint inhibitors in HCC: Cellular, molecular and systemic data. Semin Cancer Biol. 2022. 10.1016/j.semcancer.2022.01.005 35065242

[33] Jácome AA, Castro ACG, Vasconcelos JPS, Silva M, Lessa MAO, Moraes ED (2021). Efficacy and safety associated with immune checkpoint inhibitors in unresectable hepatocellular carcinoma: a meta-analysis. JAMA Netw Open.

[34] Liu Y, Ma J, Wang X, Liu P, Cai C, Han Y (2023). Lipophagy-related gene RAB7A is involved in immune regulation and malignant progression in hepatocellular carcinoma. Comput Biol Med.

[35] Qin Z, Xie B, Qian J, Ma X, Zhang L, Wei J (2023). Over-expression of RRM2 predicts adverse prognosis correlated with immune infiltrates: a potential biomarker for hepatocellular carcinoma. Front Oncol.

[36] Li D, Huang S, Luo C, Xu Y, Fu S, Liu K (2023). CKLF as a prognostic biomarker and its association with immune infiltration in hepatocellular carcinoma. Curr Oncol.

