# Supplementary material for: ATP6V1F is a novel prognostic biomarker and potential immunotherapy target for hepatocellular carcinoma
Source: BMC Med Genomics. 2023 Aug 16;16:188. doi: 10.1186/s12920-023-01624-6 (PMC10428557; doi:10.1186/s12920-023-01624-6)
Supplement: Supplementary file 1 — Additional file 1: Figure S1. The PPI network of ATP6V1F constructed using the STRING database. Figure S2. TIDE scores in ATP6V1F-low and ATP6V1F-high groups in HCC. Set the top ¼ as high expression group and the bottom ¼ as low expression group. ****P < 0.0001. G1: low expression group, G2: high expression group. Figure S3. Western blot assays verified that ATP6V1F protein expression was successfully knocked down by si-ATP6V1F in Hepg2 and SMMC7721 cells (A). Statistical results of western blot assays (B). ***P < 0.001. [file 12920_2023_1624_MOESM1_ESM.pdf]

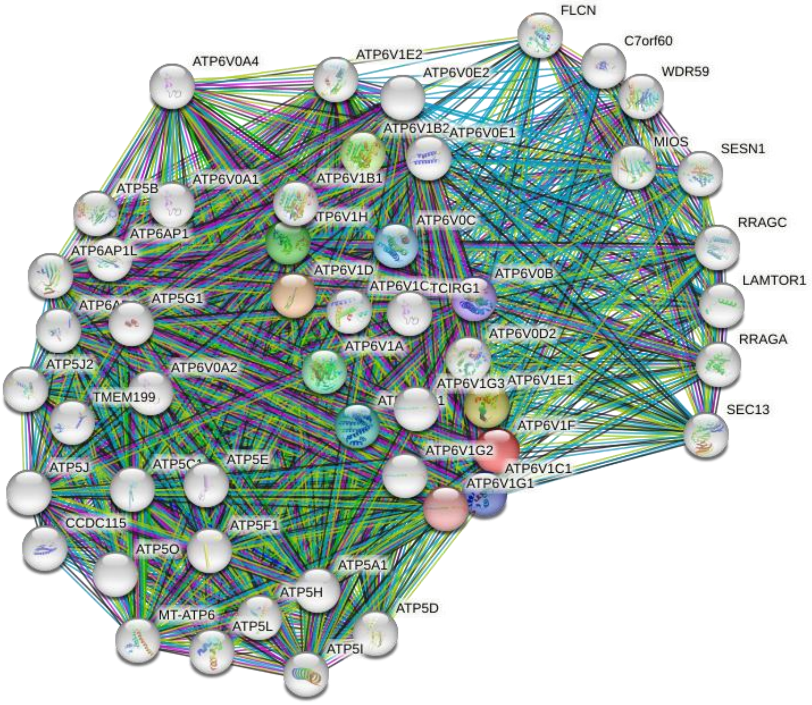

Figure S1. The PPI network of ATP6V1F constructed using the STRING database.

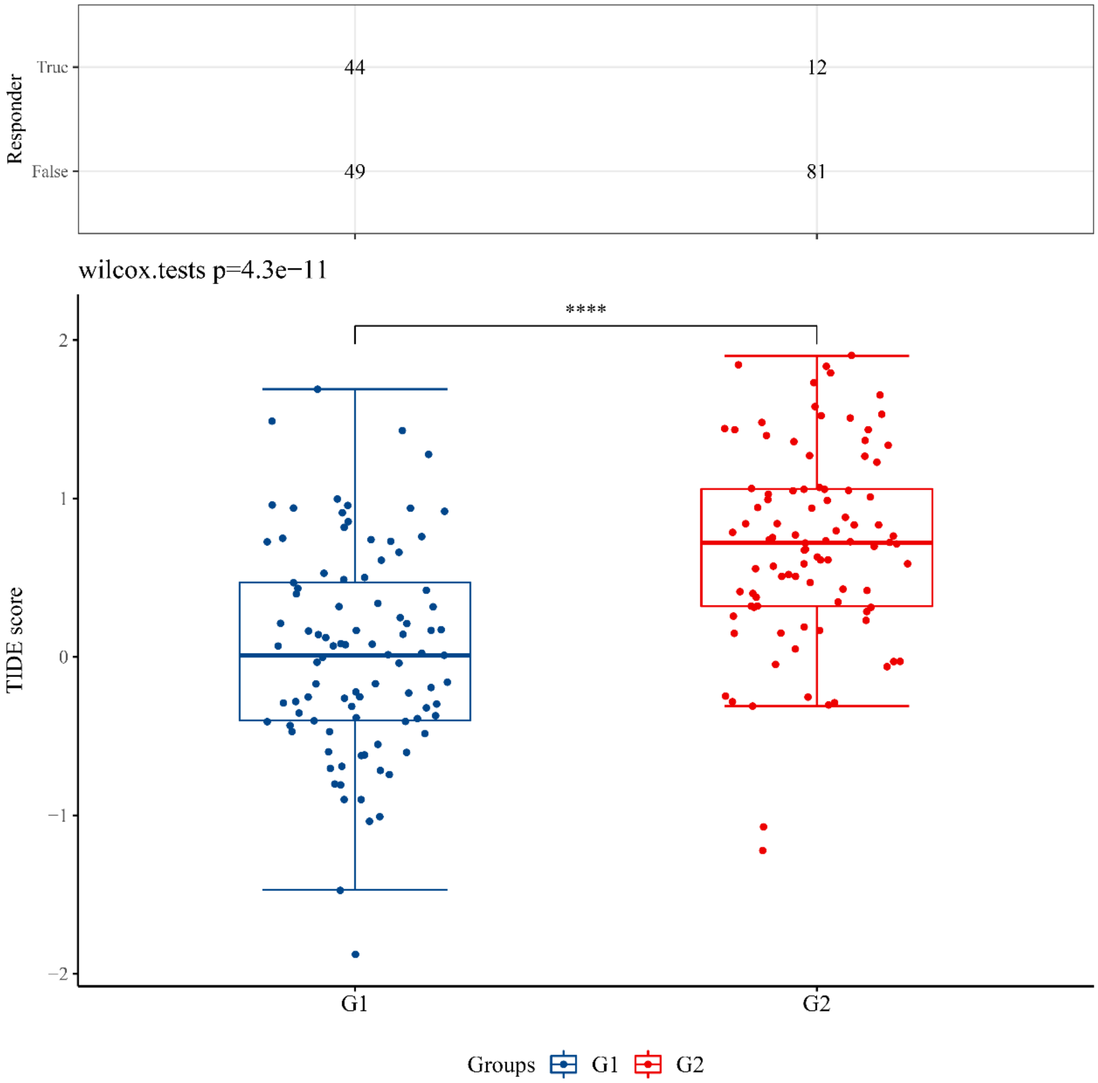

Figure S2. TIDE scores in ATP6V1F-low and ATP6V1F-high groups in HCC. Set the top ¼ as high expression group and the bottom ¼ as low expression group. ****P < 0.0001. G1: low expression group, G2: high expression group.

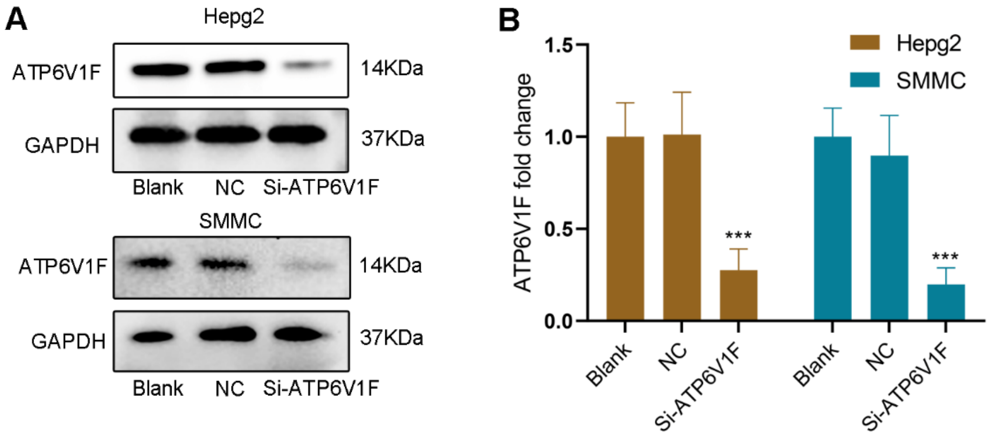

Figure S3. Western blot assays verified that ATP6V1F protein expression was successfully knocked down by si-ATP6V1F in Hepg2 and SMMC7721 cells (A). Statistical results of western blot assays (B). ***$P < 0.001$.

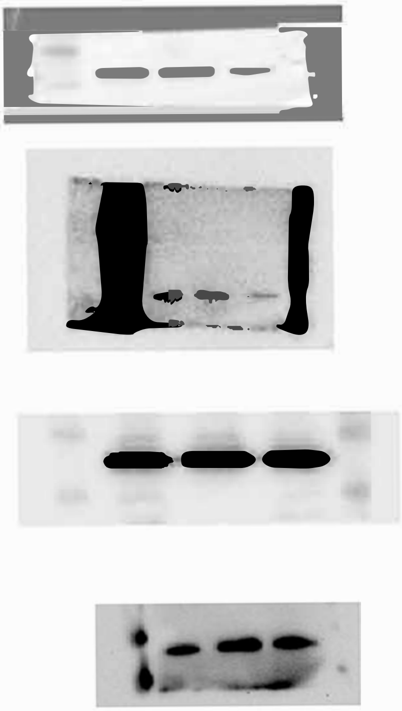

Original images of western blot assays
