# Supplementary material for: ATP6V1F is a novel prognostic biomarker and potential immunotherapy target for hepatocellular carcinoma
Source: BMC Med Genomics. 2023 Aug 16;16:188. doi: 10.1186/s12920-023-01624-6 (PMC10428557; doi:10.1186/s12920-023-01624-6)
Supplement: Supplementary file 2 — Additional file 2: Table S1. The top ranked and overlapping hub genes according to 11 topological algorithms in the PPI networks. [file 12920_2023_1624_MOESM2_ESM.zip › AddCorr1.pdf]

Dear Norman Rey

Thank you for your query. Please find attached. Please could you add an author query for them to confirm this file.

Should you have any further questions or concerns, please do not hesitate to contact me.

With kind regards,
Werner

Werner von Gruenewaldt
Project Coordinator Open Access, Production

BMC
Van Godewijckstraat 30, 3311 GX Dordrecht, The Netherlands
werner.vongruenewaldt@biomedcentral.com
www.biomedcentral.com | www.springeropen.com

From: Villafranca Norman Rey <Norman.Villafranca@straive.com>
Sent: Monday, August 7, 2023 4:14 PM
To: Werner von Gruenewaldt <werner.vongruenewaldt@biomedcentral.com>
Cc: SPi-BMCJournalsTeam <SPi-BMCJournalsTeam@spi-global.com>
Subject: BMC Medical Genomics: 12920_2023_1624
Importance: High

Title: ATP6V1F is a novel prognostic biomarker and potential immunotherapy target for hepatocellular carcinoma
DOI: 10.1186/s12920-023-01624-6
EM: 1966df53-0f37-418d-9763-f60a5084ebd6

Dear Werner,

We are currently starting the process of making a provisional pdf that is suitable for publication. We would be very grateful if you could provide missing e-file for Tale S1 since mentioned in the manuscript.

Please send the needed file via email as soon as possible so we can proceed with the article.

Thank you.

Kind regards,

Norman Rey D. Villafranca
Project Officer
Straive
https://www.straive.com

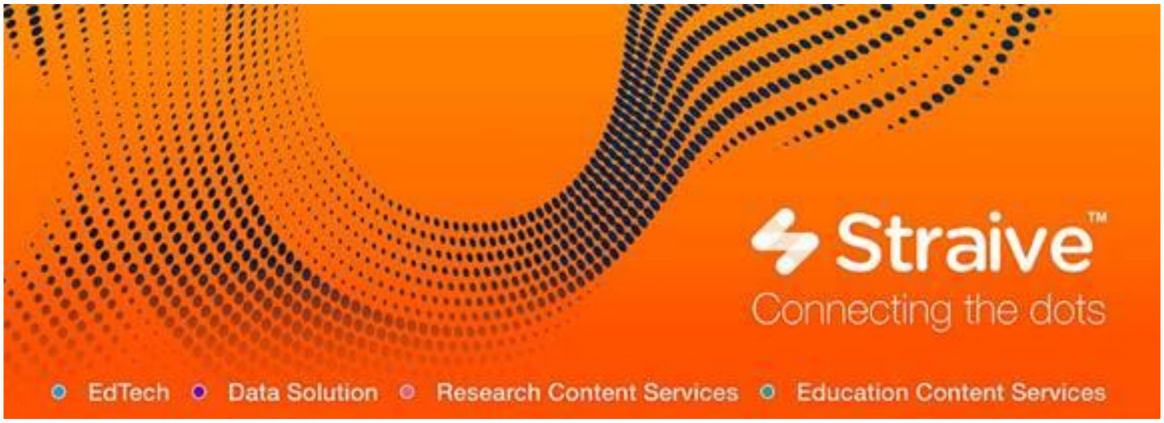

CONFIDENTIALITY NOTICE: This email, including its attachments, is intended for the use of the person/s it is addressed to. It may contain personal data, or information that is protected or privileged, which are protected from unauthorized use or disclosure by law.

If you are not the intended recipient, any dissemination, retention or use of any information contained in this email is prohibited. If you have received this email in error, please promptly notify the sender by reply email and delete the original email and any backup copies without reading them.

If you have questions or clarifications regarding any matter relating to data protection, you may write to the Straive Data Protection Office at dpo@straive.com. You may also file a complaint or report a security incident involving personal data by writing to: dpo@straive.com

---

DISCLAIMER: This e-mail is confidential and should not be used by anyone who is not the original intended recipient. If you have received this e-mail in error please inform the sender and delete it from your mailbox or any other storage mechanism. Biomed Central Limited does not accept liability for any statements made which are clearly the sender's own and not expressly made on behalf of Biomed Central Limited or one of their agents.
Please note that Biomed Central Limited and their agents and affiliates do not accept any responsibility for viruses or malware that may be contained in this e-mail or its attachments and it is your responsibility to scan the e-mail and attachments (if any).
Biomed Central Limited. Registered office: The Campus, 4 Crinan Street, London, N1 9XW. Registered Number: 03680030 England.
