# Supplementary material for: ATP6V1F is a novel prognostic biomarker and potential immunotherapy target for hepatocellular carcinoma
Source: BMC Med Genomics. 2023 Aug 16;16:188. doi: 10.1186/s12920-023-01624-6 (PMC10428557; doi:10.1186/s12920-023-01624-6)
Supplement: Supplementary file 2 — Additional file 2: Table S1. The top ranked and overlapping hub genes according to 11 topological algorithms in the PPI networks. [file 12920_2023_1624_MOESM2_ESM.zip › Table S1.docx]

**Table S1.** **The top ranked and overlapping hub genes according to 11 topological algorithms in the PPI networks**

| ID | Name | Differential expression in HCC and normal tissues | Relationship with the OS of HCC |
| --- | --- | --- | --- |
| ATP6V1A | ATPase H+ transporting V1 subunit A | non-significant | non-significant |
| ATP6V1B1 | ATPase H+ transporting V1 subunit B1 | non-significant | non-significant |
| ATP6V1E1 | ATPase H+ transporting V1 subunit E1 | Higher in tumor | negative correlation |
| ATP6V1G1 | ATPase H+ transporting V1 subunit G1 | non-significant | non-significant |
| ATP6V1C1 | ATPase H+ transporting V1 subunit C1 | Higher in tumor | non-significant |
| ATP6V1F | ATPase H+ transporting V1 subunit F | Higher in tumor | negative correlation |
| ATP6V1D | ATPase H+ transporting V1 subunit D | Higher in tumor | correlation |
| TCIRG1 | T cell immune regulator 1, ATPase H+ transporting V0 subunit a3 | non-significant | non-significant |
| ATP6V1C2 | ATPase H+ transporting V1 subunit C2 | non-significant | non-significant |
| ATP6V1G3 | ATPase H+ transporting V1 subunit C3 | non-significant | non-significant |

The differential expression of genes in HCC and normal tissues and the correlation between gene expression and OS in HCC patients were obtained from the GEPIA website. p-value < 0.05 was regarded as significant.
